# FunTree: advances in a resource for exploring and contextualising protein function evolution

**DOI:** 10.1093/nar/gkv1274

**Published:** 2015-11-20

**Authors:** Ian Sillitoe, Nicholas Furnham

**Affiliations:** 1 Institute of Structural and Molecular Biology, University College London, Darwin Building, Gower Street, London WC1E 6BT, UK; 2 Department of Pathogen Molecular Biology, London School of Hygiene and Tropical Medicine, Keppel Street, London WC1E 7HT, UK

## Abstract

FunTree is a resource that brings together protein sequence, structure and functional information, including overall chemical reaction and mechanistic data, for structurally defined domain superfamilies. Developed in tandem with the CATH database, the original FunTree contained just 276 superfamilies focused on enzymes. Here, we present an update of FunTree that has expanded to include 2340 superfamilies including both enzymes and proteins with non-enzymatic functions annotated by Gene Ontology (GO) terms. This allows the investigation of how novel functions have evolved within a structurally defined superfamily and provides a means to analyse trends across many superfamilies. This is done not only within the context of a protein's sequence and structure but also the relationships of their functions. New measures of functional similarity have been integrated, including for enzymes comparisons of overall reactions based on overall bond changes, reaction centres (the local environment atoms involved in the reaction) and the sub-structure similarities of the metabolites involved in the reaction and for non-enzymes semantic similarities based on the GO. To identify and highlight changes in function through evolution, ancestral character estimations are made and presented. All this is accessible through a new re-designed web interface that can be found at http://www.funtree.info.

## INTRODUCTION

In order to understand how proteins have evolved to perform their function requires bringing together diverse data ranging from protein sequences and structures through functional descriptors, such as reaction chemistry and mechanistic properties of enzymes. Bringing together this information is crucial in the light of the continuous flood of genomic data as insights into the evolution of protein function provide one of the best routes for predicting functions of uncharacterised proteins (1). Few resources currently bring together in-depth analysis of evolutionary history with relationships in protein function (e.g. metabolite characteristics) or take connections between overall reactions into consideration (e.g. similarities between reaction centres, bond order changes and sub-structures). A number of studies have been undertaken on collections of protein superfamilies whose membership predominantly consists of enzyme structures and sequences and numerous studies on single superfamilies (2–4).

To meet this challenge FunTree (5) was developed to bring together protein structures from the CATH (6) classification of domains, sequences from UniProt (7) and CATH-Gene3D (8), as well as functional and chemical information from a variety of sources including the manually curated MACiE (9) and Catalytic Site Atlas (CSA) (10) databases. Focused on enzyme function evolution, it catalogued 276 enzyme containing structurally defined domain superfamilies. Here we present an update to FunTree that has expanded to cover all types of protein function, not just enzymes, in over 2,340 CATH superfamilies.

## EXPANDING THE DATASET

**Figure 1.**
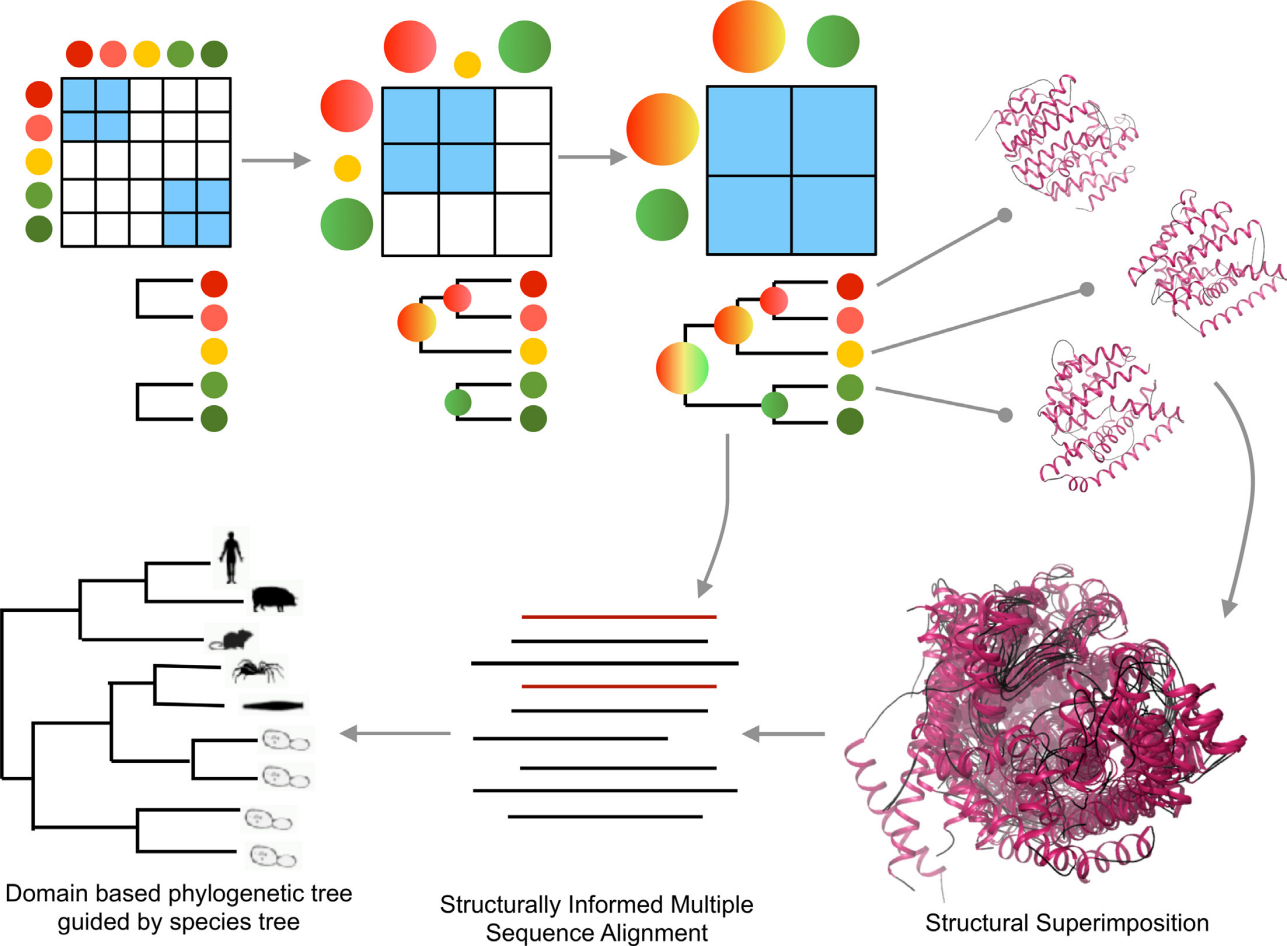
An overview of the protocol for using FunFams derived from a new agglomerative clustering technique combined with structural superimpositions to generate a structurally informed multiple sequence alignment that can be used in the phylogenetic analysis. Pairs of most similar sequences (colored circles) are aligned and iteratively pairs of most similar alignment profiles are aligned. Structures associated with sequences contained within the agglomerative profiles are structurally aligned. Based on this alignment the other sequences within the profile are integrated into the structural alignment based on the fixed profile.

The first version of FunTree focused on 276 enzyme containing structurally defined domain superfamilies. Due to difficulty in aligning and superimposing all domains within large, structurally variable superfamilies imposed by the many structural decorations outside the common structural core, FunTree used a method to group structural domains and augment them with domain sequences from CATH-Gene3D to form structurally similar groups (SSGs). It used a combination of CORA, Profit (Martin, A.C.R. and Porter, C.T., http://www.bioinf.org.uk/software/profit/), Blastp and FUGUALI (also part of the FUGUE (11) software) to generate robust structurally informed multiple sequence alignments of structurally similar groups (SSGs) that could be used in the subsequent phylogenetic analysis. To bring more structures and sequences together and to reduce the number of SSGs a new clustering and alignment method was implemented. In summary, structurally coherent relatives are defined as those that superpose within 9Å RMSD. CATH identifies SSGs comprising relatives within a superfamily clustering within this threshold. Structural representatives from across the superfamily were selected from CATH functional families. Functional families (FunFams) are identified within each superfamily using a novel agglomerative clustering method (12) that groups sequences sharing similar sequence patterns that relate to specificity determining positions in the family. Using CORA (13) to generate structural superimposition of the structural representative, the FunFam profiles for each structural representative are integrated into the structural alignment using MAFFT (14) to generate a structurally informed multiple sequence alignment. A summary of this new protocol is shown in Figure 1.

In employing this new protocol the average number of SSGs within a superfamily has reduced, while the number of sequences and structures that can be included within an alignment has greatly increased as the method allows for remoter homologues to be included. This is a result of our ability to confidently associate sequences with each other and to confidently align then through structural representatives. This has resulted in alignments that contain very large number of sequences (thousands or even tens of thousands). To make the phylogenetic analysis tractable and the resulting trees easy to view and navigate a filtering algorithm has been implemented to select suitable representatives of subsequent analysis. The program attempts to optimise the number of sequences to less than 600 while still maximising phylogenetic diversity, structural coverage and novel functional representation. Many SSGs though do not require any filtering and all sequences and structures are included. Overall these changes have allowed 2340 superfamilies to be processed.

## COMPARING PROTEIN FUNCTIONS AND ANCESTRAL CHARACTER ESTIMATION

One of the major additions to FunTree is the inclusion of Gene Ontology (GO) annotations (15). These are taken from the sequence's UniProt record where the record has been reviewed by a curator and has extended FunTree from not only looking at novel function evolution for enzymes but to all types of protein function. GO term identifiers are displayed, like the enzymes Enzyme Commission (E.C.) number (16) on the tree and linked to GO or IntEnz (17) respectively for full descriptions of the terms. For enzymes the E.C. number is displayed in preference to the GO term.

As well as displaying functional annotations, comparisons of the functions are made. For GO annotations, a semantic similarity measure is made using the Wang method implemented in the GOSemSim R package (18). An all-by-all similarity matrix is calculated for each GO term associated with a sequence within the SSG. The results are clustered using a hierarchical clustering method implemented in the PVClust package in R (19). The results are displayed on the tree as a colored circle shown at the leaves of the tree, where the color is graduated so that similar colors have similar functional similarities and very different colors have very different functional similarities.

It is important to note that both E.C. and GO annotations are made at the level of the gene product, though the functional unit they are describing are often smaller, frequently comprising of a single domain. Though domains are regularly considered to be the functional unit, more complex functional units composed of multiple domains or gene products (molecular machines) exist. As FunTree is based on CATH structural domains, E.C. and GO terms may be ascribed to a domain derived from the gene product and therefor may not contribute exclusively to that function and care needs to be taken when analysing these ‘confusion’ domains.

**Figure 2.**
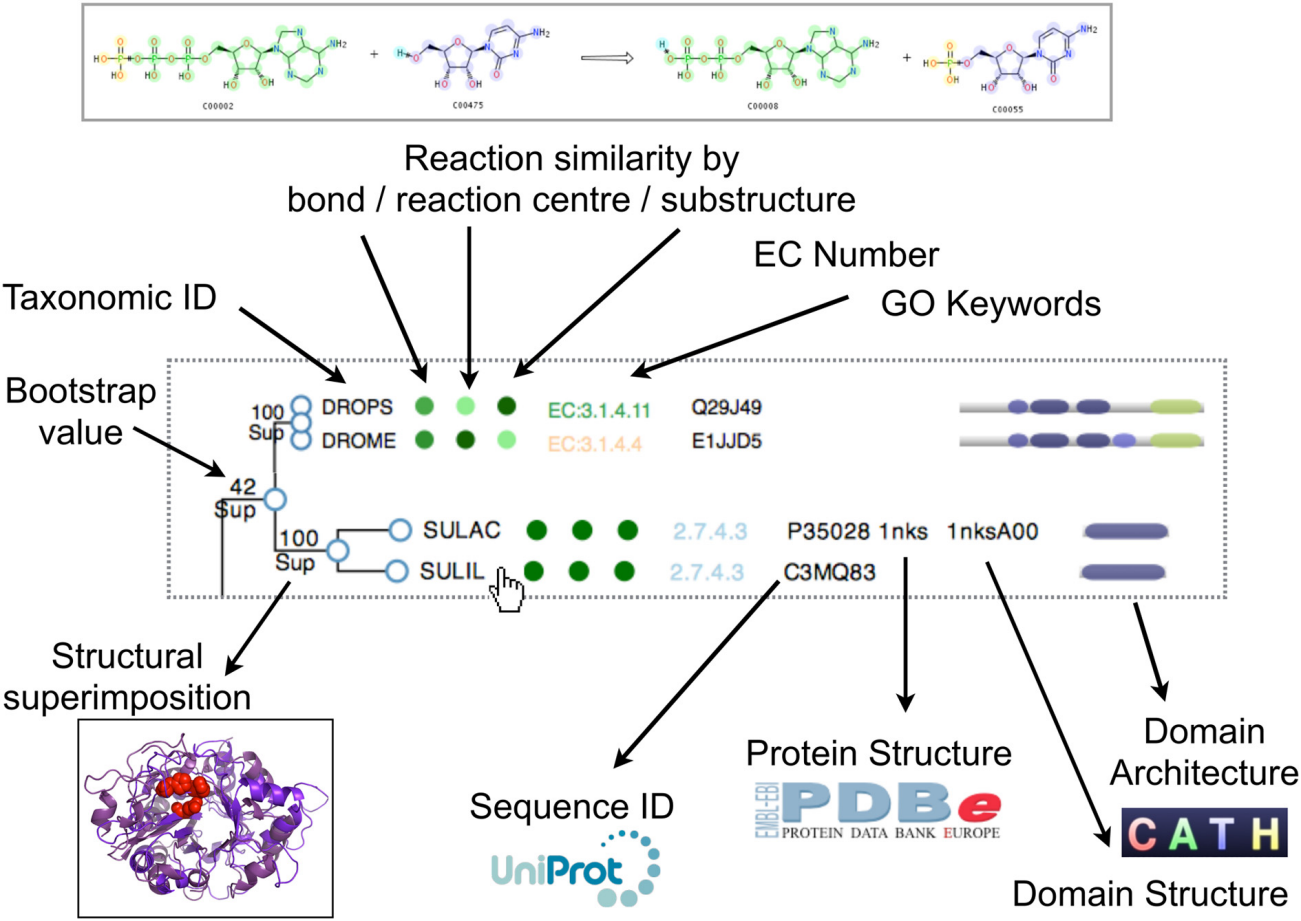
An overview of the data presented at the leaves of the trees within FunTree.

For enzymes the overall reactions are compared using the IUBMB reactions describing each of the E.C. numbers. Like the GO annotations, each reaction is compared to each other within a SSG using the EC-Blast algorithm (20). Briefly, this uses atom-atom mapping to determine bond changes and reaction patterns, using a variation of the Dugundji-Ugi matrix model. Comparisons are made using three types of normalized similarity scores. The first, bond order, compares the changes in the number and type of bonds that are being broken and formed. Secondly, the reaction centre metric compares the local chemical environment around the centre of the reaction, i.e. the atoms covalently linked to the atoms forming the bond that is broken/formed in a reaction. Thirdly, the substrates and products of the reactions are compared using a common sub-graph detection algorithm. This is in addition to the comparisons of metabolites made in the original FunTree, though this is now only displayed though a separate metabolite similarity tree as a cumulative measure is made by the third type of EC-Blast similarity measure. Again, similar to how GO similarities are displayed, each of the EC-Blast similarity measures are displayed at the leaves of the tree colored by the level of similarity to each other within the tree. A summary of the information contained at the leaves of a tree is shown in Figure 2.

**Figure 3.**
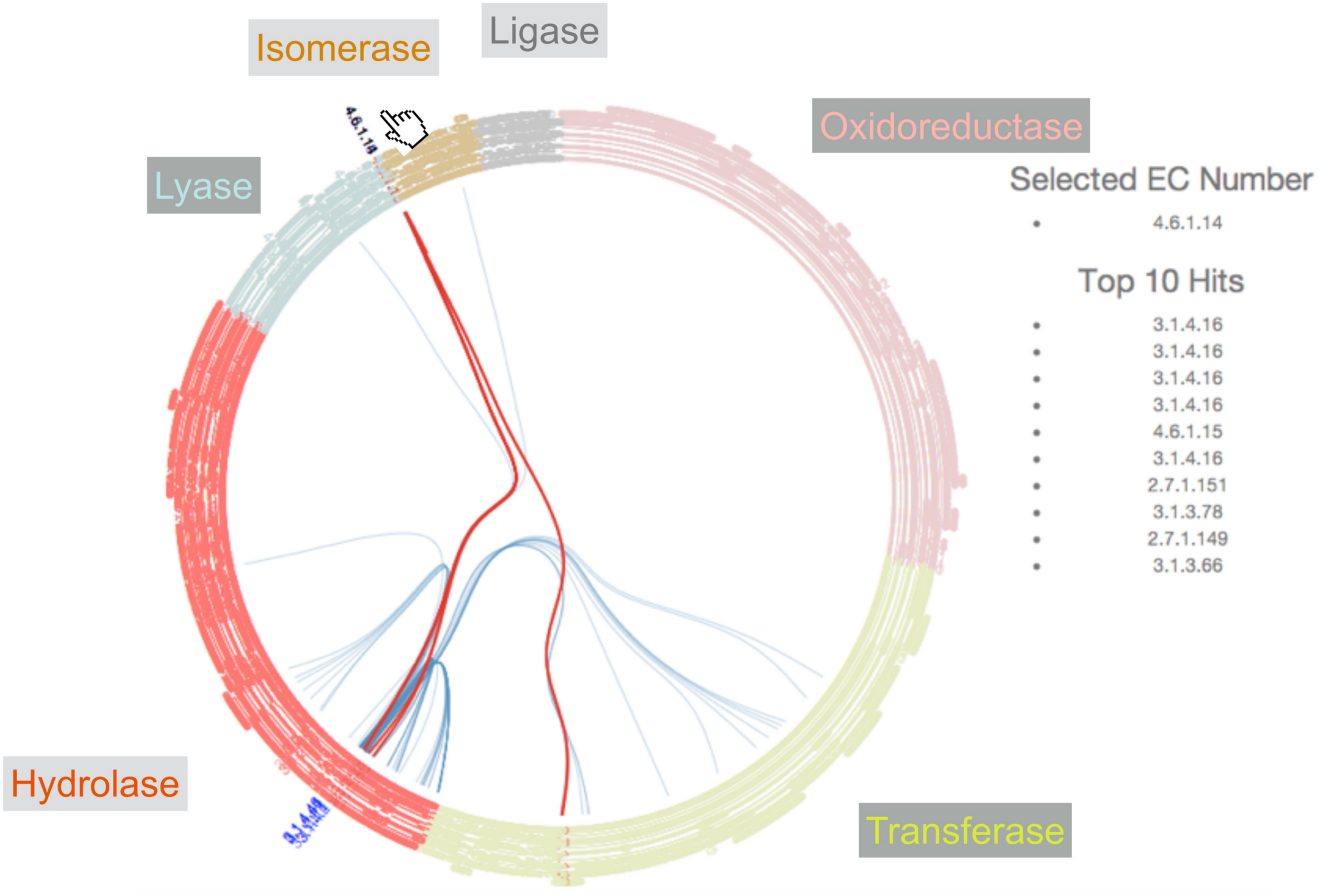
The interactive visualization of the relationships between functions present in the superfamily related to the top 10 most similar reactions. The E.C. classification is rendered as circular rooted tree, with the leaves are colored by primary E.C. class. E.C. numbers that are found in the superfamily are pushed out of the circle and are colored blue. Links between these and their 10 most similar functions as calculated by EC-Blast are highlighted in blue, tracing the path through the tree between them. By hovering over a function within the superfamily, highlights in red the connections to the top 10 most similar reactions, which are also listed on the right.

In addition to calculating the all-by-all reaction similarities within an SSG, EC-Blast has pre-calculated the all-by-all similarities of all known biochemical reactions and has implemented a method for ranking the most similar reactions to a given reaction. At the superfamily level, a new visualization of the results of this ranking is displayed showing the relationships between the top ten hits for each of the reactions (as represented by an E.C. number) present in the superfamily. An example of this visualization is shown in Figure 3.

**Figure 4.**
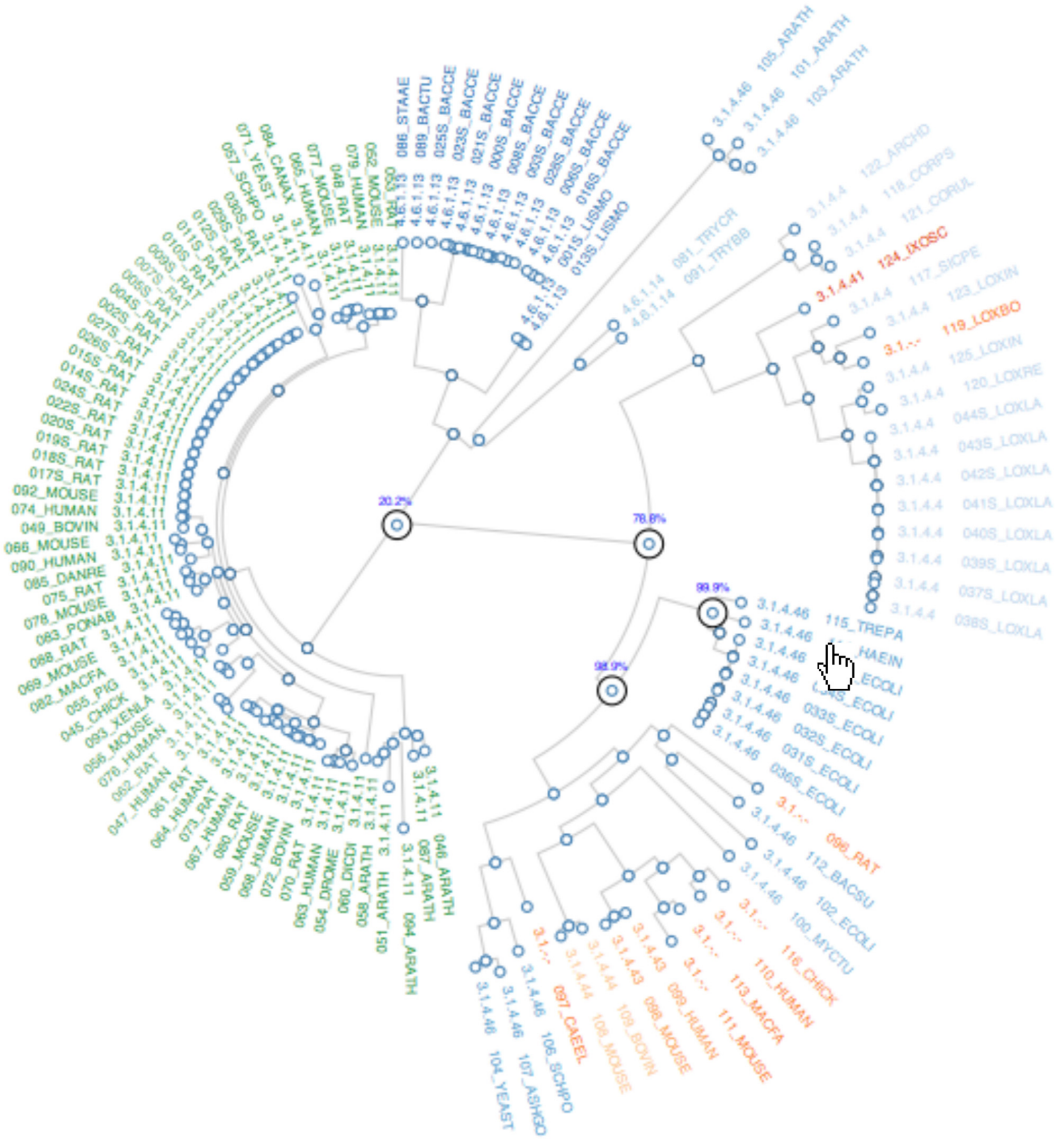
The interactive visualization showing the results of the ancestral character estimation. The tree is rendered as a circular rooted tree. By hovering over the functional annotation at the leaf of the tree, the ancestral nodes from the leaf back to the root are highlighted with the percentage likelihood values for that function at each node. By hovering over a node the function that has the maximum likelihood value is displayed.

As well as displaying sequence functions and their similarities, the functional annotations are used to calculate the ancestral function for each node within the tree. Leaves without a function annotation are pruned from the tree. Ancestral character estimations are made using the discrete ancestral character estimation algorithm with an equal rates model as implemented in the APE package in R (21). At each node in the tree, maximum likelihood estimation is made of the most probable function. It should be noted that the ancestral function is assumed to be one of the modern functions observed at the leaves of the tree. This permits the functional changes from parent node to a child node to be traced through the tree and to catalogue the changes in function based on the EC number. The results of this are displayed in an interactive visualisation (Figure 4).

## UPDATED DATA PRESENTATION AND NAVIGATION

In expanding and improving FunTree, the website has been completely redeveloped. To make all the data visualizations interactive and responsive, data is served as JSON objects and rendered on the client side. It uses the D3 javascript libraries (http://d3js.org) to generate a number of improved and new visualizations. As well as the new data views described in the previous sections, the phylogenetic trees that lie at the heart of FunTree have been improved. Maintaining the zoom, panning and hyperlinking functionality found in the original FunTree that rendered the trees using the GoogleMaps API, nodes can now be collapsed and expanded, new data can be easily added to the leaves and a improved and more accurate cartoon of the multi domain architecture has been added.

At the superfamily level, an interactive web interface emulating ArchSchema (22) to visualize relationships in multi-domain architecture has been implemented. Using a force-directed graph, it self organizes a graph of MDAs present in the superfamily, where nodes are unique MDAs, connected by increasing complexity of the domain architecture (centred on the MDA that consists of the single superfamily domain). Also at the superfamily level, the E.C. hierarchy is rendered as a rooted circular tree. Nodes and branches that represent the E.C. numbers present in the superfamily are highlighted.

**Figure 5.**
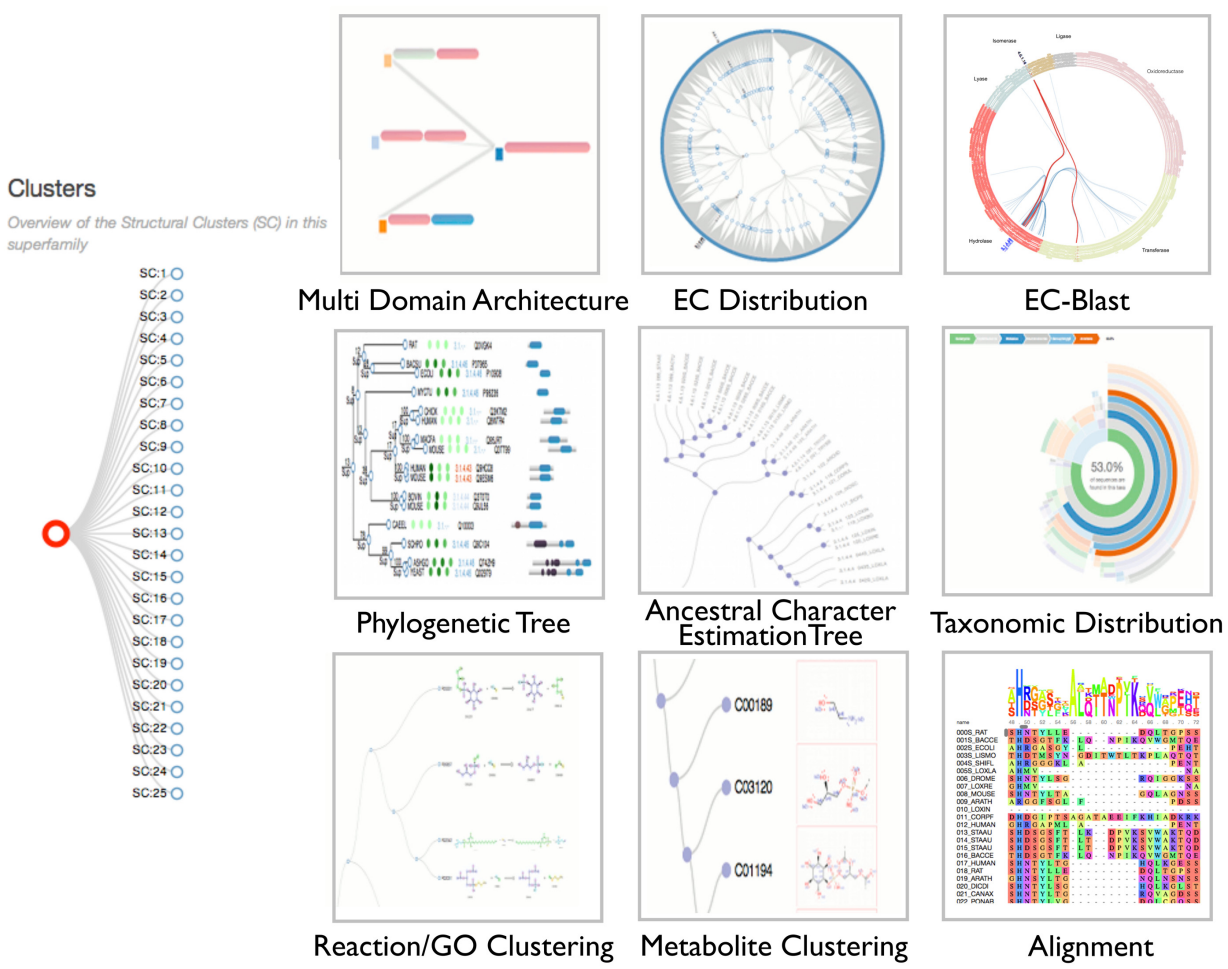
An overview of the tiles presented within FunTree that link to the various data visualizations. At the superfamily level, the multi-domain architectures, EC-distribution and EC-Blast results are available. At the SSG level the phylogenetic tree, ancestral character estimations, taxonomic distribution, functional clustering tree and alignments are available. On the left, the interactive browser to move between superfamily and SSG levels.

At the SSG level a number of new data views have been developed including trees that show the relationships in similarity of the reactions, GO terms and metabolite similarities of the functions catalogues within the SSG. Additionally, the taxonomic composition of the sequences presented in the tree is shown as a radial plot displaying the taxonomic lineage and relative abundance of the taxa class. Finally a new display of the multiple sequence alignment using the BioJS (23) module is presented. This allows, amongst other things, the user to highlight the alignment as desired using a range of coloring schemes, a consensus sequence logo to be displayed and an alignment overview window, which can be used to zoom into a particular part of the alignment. An overview of the new interface is shown in Figure 5.

## SUMMARY

The recent developments in FunTree presented here have expanded the number of superfamilies analyzed to cover most of the superfamilies defined in CATH. By including GO annotations as well as calculating ancestral character estimations, it is possible to investigate the evolution of novel functions across all types of protein function. The addition of different measures of functional similarity, especially the novel measures of reaction similarity, allows the user to gauge how big a change has occurred between functions. In the context of enzymes, displaying the most similar reactions can provide insights into new functions that could be performed by members of the superfamily but have yet to be observed. Being able to explore and contextualize the evolution of protein function provides insights that will be useful in predicting the function of uncharacterized sequences as well as the design of new synthetic enzymes.
